# Let’s Be Realistic with Rurality: Timely Surgical Management of Open Fractures in Rural and Remote Patients

**DOI:** 10.3390/jcm15124516

**Published:** 2026-06-11

**Authors:** Travis Jennings, Katie Wang, William G. Blakeney, Nicholas Calvert

**Affiliations:** 1 Department of Orthopaedics, Royal Perth Hospital, Perth, WA 6000, Australia; travis.jennings@health.wa.gov.au (T.J.); katie.wang@health.wa.gov.au (K.W.); nicholas.calvert@health.wa.gov.au (N.C.); 2 Faculty of Medicine, Surgery, University of Western Australia, Perth, WA 6009, Australia

**Keywords:** orthopaedic surgery, rural medicine, open fracture, trauma

## Abstract

**Background:** Rurality in Western Australia presents challenges in the management of open fractures for the state’s trauma centre. Open fractures are associated with high morbidity, and the BOA Standards for Trauma and Orthopaedics (BOAst) guidelines recommend timelines for surgical management to minimise complications. This study aimed to describe the timelines to surgical management for rural patients with severe open fractures and to identify system-level factors contributing to delays. **Methods:** A retrospective single-centre study was conducted on all rural patients with open fractures requiring fixation and soft-tissue coverage between January 2020 and December 2023. Data was collected from the trauma registry and electronic medical records, including injury characteristics, transfer details, timing of surgical management, and complications. **Results:** Fifteen rural patients met the inclusion criteria. The median time to initial debridement from first healthcare presentation was 27.6 h. The mean transfer time to the trauma centre was 14.5 h, and the median time to definitive fixation and soft tissue coverage was 159.7 h. Identified modifiable factors to minimise delays on arrival to the trauma hospital include prioritisation of rural patients at the time of arrival, accessibility to an orthoplastics theatre, and minimising the total number of debridements prior to definitive reconstruction. **Conclusions:** Substantial delays in the surgical management of rural patients with open fractures were observed, largely related to interhospital transfer and system-level factors at the tertiary centre. These delays limit the feasibility of compliance with the BOAst guidelines and highlight the need for context-specific strategies to improve the timely care of this high-risk population.

## 1. Introduction

Western Australia’s (WA) geographic barriers present challenges in the timely management of open fractures. Open fractures are a complex wound pattern associated with a high risk of morbidity, mortality, and postoperative complications [1].

The British Orthopaedic Association Standards for Trauma and Orthopaedics (BOAst) guidelines outline standards of care for the management of open fractures [2]. Within the BOAst guidelines are recommended timelines for (1) initial debridement and (2) definitive fixation and soft tissue coverage. The recommendations for time to initial debridement were based on a proposed injury classification. Type A injuries were recommended to be debrided immediately and included highly contaminated wounds or neurovascular compromise; type B injuries were to be debrided within 12 h and included high-energy wounds without characteristics of type A injuries; and type C injuries were to be debrided within 24 h and included low-energy injuries. The recommended timeline for definitive fixation and coverage was within 72 h of injury for all three injury types.

WA faces unique challenges due to its large geographical size and the centralisation of tertiary healthcare services to the Perth metropolitan area. As a result, patients from rural and remote regions often experience long journeys (and associated delays) to reach appropriate healthcare [3]. The state of WA encompasses approximately 2.6 million square kilometres, extending over 2500 km between its furthest points [4]. A significantly larger landmass compared to that of the United Kingdom (approximately 243,000 square kilometres, one tenth the size of WA), where the BOAst guidelines were developed. Patients residing outside of the metropolitan area requiring interhospital transfer frequently encounter significant delays to surgical fixation, often exceeding 24 h [5]. Patients from remote communities, which are mostly populated by people of Indigenous Australian descent [6], are often the most affected due to their journey requiring multiple staged transfers between regional hubs prior to arriving in Perth. Transfer times are partially dependent on the assigned clinical priority by the Royal Flying Doctor Service (RFDS). A previous study reported a mean transfer time of 11.6 h for major trauma patients in WA (Injury Severity Score (ISS) > 15) [7]. Rural major trauma patients have also been shown to present with a higher median ISS and have an increased mortality risk (odds ratio 2.60) compared with metropolitan patients [8]. However, it is important to note that not all patients with open fractures meet the criteria for major trauma designation and may accordingly be assigned a lower clinical priority for transfer.

The combination of high patient volume, extensive geographical coverage, and extreme remoteness of many communities across WA contributes to delays in the timely management of open fractures. The delays experienced by rural patients in accessing time-critical surgical intervention have the potential to expose this cohort to worsened clinical and functional outcomes. This study aims to describe timelines to surgical management for rural patients with severe open fractures in WA and to identify system-level contributors to delay, and whether the BOAst guidelines are realistic for the management of open fractures in this patient cohort.

## 2. Methods

### 2.1. Patient Cohort

A retrospective single-centre cohort study was conducted to review outcomes in patients with open fractures requiring soft tissue flap coverage. Data was gathered from a trauma registry of patients admitted to a tertiary trauma centre in Perth, Australia, with open fractures requiring flap coverage from January 2020 to December 2023.

### 2.2. Inclusion and Exclusion Criteria

The patients included were above the age of 16 and had an open limb fracture that required transfer from a rural health setting, and received flap soft tissue coverage with at least 6 months of follow-up. The minimum follow-up period was allocated to allow adequate time for monitoring of delayed complications. The exclusion criteria were patients requiring a delayed flap due to wound breakdown, as this would incorrectly represent timeframes, or patients receiving a primary amputation.

### 2.3. Data Collection

The patient’s records, operation reports, discharge summaries and clinic letters were reviewed to collect demographic information and data points relevant to inpatient management and postoperative outcomes. Time to surgical intervention was measured from the time of first patient presentation to a rural emergency health care facility. It was not possible to measure from the time of injury due to incomplete documentation and the inaccuracies inherent to the timing of events pre-hospital. Transfer time was the time from presentation at the rural health centre to the time of arrival at the trauma emergency centre. The complications recorded included deep infection (requiring inpatient management, repeat debridement or prolonged antibiotics), fixation failure (aseptic non-union requiring repeat surgery) or flap failure (flap necrosis requiring flap revision). Complications were further categorised as acute, occurring within the index admission or delayed, occurring after discharge from initial admission to the trauma centre.

### 2.4. Statistical Analysis

Retrospectively collected data were managed with Microsoft Excel (version 2605; Redmond, WA, USA). Given the small sample size, no inferential statistical testing was planned, and results are presented descriptively. Continuous variables were presented as the mean and standard deviation (SD), or by the median and inter-quartile range for skewed continuous data. Categorical variables were presented as counts and percentages.

### 2.5. Objectives

The aim of this study is to (1) quantify the time to initial debridement and definitive fix and flap experienced by rural patients, and (2) evaluate whether the BOAst guidelines are appropriate for rural and remote WA. The secondary aim of the study was to evaluate modifiable factors contributing to delays and the rate of complications.

## 3. Results

### 3.1. Demographics

Fifteen patients were extracted from the trauma registry who were transferred from a rural healthcare setting with an open limb fracture requiring flap coverage from January 2020 to December 2023 (Table 1). Four patients sustained type A injuries (due to being highly contaminated, no reported injuries included neurovascular compromise), and the remaining eleven had type B injuries. The average straight-line distance travelled by rural patients to Perth was 772 km, the furthest being 1934 km. The patient’s sex, age, Indigenous status, injury severity score (ISS), and injury classification were collected (Table 1). The average age of patients was 33 years, and a high proportion were Indigenous (46.6% of the patient cohort, compared to 3.8% of the Australian population) [6]. The median ISS of the patient cohort was 13 (Interquartile Range (IQR) 10–22). All injuries were caused by motor vehicle accidents: nine resulted from automobile accidents (60%), four from motorcycle accidents (26.7%), and the remaining two involved pedestrians struck by automobiles (13.3%).

### 3.2. Time to Trauma Centre from Rural Centre

The average time to transfer from rural healthcare presentation to the emergency department of the trauma centre was 14.5 h. This included patients transferred via aeroplane, ambulance, and helicopter.

### 3.3. Time to Debridement

**Figure 1:**
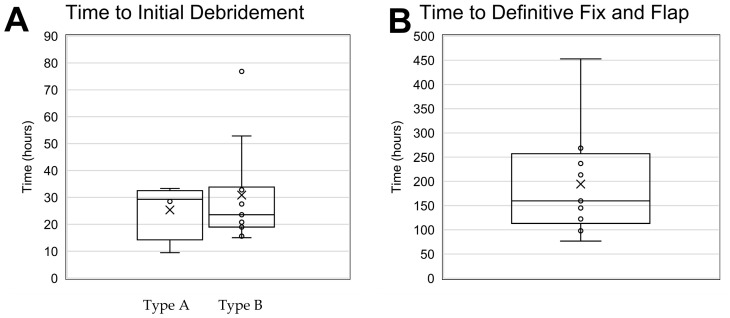
(**A**) Time to initial debridement from rural healthcare setting (by injury subgroup); (**B**) time to definitive fix and flap from rural healthcare setting.

The median time to first debridement in the patient cohort was 27.55 h (IQR: 19.9–33.075), with mean times of 25.4 h and 30.9 h for injury groups A and B, respectively. None of the rural patients underwent debridement within the BOAst-recommended timeframes (Figure 1).

### 3.4. Time to Fix and Flap

The median time to fix and flap from initial healthcare presentation was 159.7 h (IQR: 122.4–245.5), an average time of 258.8 and 165.9 h for injury groups A and B, respectively. That is, 7.7% (1/13) of patients complied with the BOAst guidelines recommendation of definitive fixation and coverage within 72 h from injury. Two patients proceeded to delayed amputation and did not receive a fix and flap procedure (one type A injury and one type B injury)—they were included in the audit as they had injuries that were deemed to require flap coverage; however, they did not proceed to the operation due to the development of neurovascular compromise prior to the operation (and post initial debridement).

### 3.5. Delays in Surgical Management

Modifiable factors contributing to rural patient delay to surgical treatment were identified, including the timing of patient operation, theatre availability for an orthoplastics combined case, and the number of debridements prior to definitive fixation and coverage. No rural patients received their initial surgical debridement outside of normal operating hours; 47% received their debridement in the morning, and 30% of these were the first “golden” case of the day. Most of the fix and flap procedures were performed on Tuesday, in line with the designated theatre session for combined cases. The average number of debridements before definitive fixation and flap coverage was 2.4. There was a strong correlation between the number of debridements and time to fix and flap (R^2^ = 0.724).

### 3.6. Complications

Deep infection was the most common acute complication (Table 2), occurring in three patients (20%; two type A injuries and one type B injury), and flap failure in the acute setting occurred in one patient (type A injury). Within the minimum 6-month follow-up, one patient was complicated by a delayed deep infection (6.7%; type B injury). Fixation failure was not reported in any patients in the acute or delayed setting.

## 4. Discussion

This study demonstrates substantial delays in the surgical management of rural patients with severe open fractures in Western Australia, suggesting that adherence to the BOAst-recommended timelines for surgical management of open fractures is challenging for this patient population. Transfer time from rural healthcare services to the tertiary trauma centre represents a critical limiting factor, with a mean duration of 14.5 h. Consequently, patients presenting with Type A and Type B open fractures exceed recommended debridement timeframes before arrival at the tertiary centre. While delays to surgical intervention are not unique to rural patients, and there are system-based issues that impact all patients regardless of rurality (such as access to operating theatres and staffing), the additional steps in the pre-hospital journey of rural patients compound this delay. This prolonged inter-hospital transfer interval constitutes a system-level challenge to guideline compliance and may expose the rural patient cohort to disproportionately higher risk of complications and poorer clinical outcomes.

Rural patients frequently arrive outside of standard operating hours due to the nature of the time of injury and travel time from rural locations. Accordingly, rural patients can experience further delays upon arriving at the trauma centre, as they often must wait until daylight hours to proceed to the operating theatre. In the patient cohort of this study, 8/15 patients arrived at the trauma hospital emergency department out of hours, none of whom were operated on out of standard hours.

However, recent studies have challenged the traditional paradigm of urgent debridement within six hours, finding that debridement performed during daylight hours by an experienced surgical team yields comparable results to debridement performed on arrival outside of standard hours [9,10]. A balance must be struck between the potential gains of earlier debridement and the potential costs and risks of out-of-hours surgical intervention. There are certainly types of injuries, such as open fracture dislocations [11], or open fractures associated with vascular injury [12], where urgent surgical intervention (including out-of-hours) is required. However, the ability to operate out of hours is often limited by staffing and the availability of operating theatres.

On this basis, a patient-specific approach is required when deciding whether to operate out-of-hours (or not). This approach, however, is likely to reduce adherence to the BOAst recommended timelines. Prioritising rural patients to morning theatre lists or first case slots can help mitigate any further delays.

The primary purpose of timely surgical management of open fractures is to optimise outcomes and reduce complication rates. One of the more common and problematic complications is deep infections [13]. Postoperative complications can have a profound impact on the individual as well as impose a substantial cost to the healthcare system, with deep infections being found to increase mortality [14], risk of delayed amputation [15], hospital length of stay [16], and lower functional outcome scores [17]. The reported rate of deep infection in the recent literature varies greatly, ranging from approximately 11% to 25% [6,7]. The total deep infection rate of 26.7% (4/15) in our audit underscores the clinical consequences of delayed surgical management. Although an association between delays to surgical intervention and infection rates was demonstrated in our study, it lacked the statistical power (due to the small cohort size) to imply causality. However, this finding is consistent with well-established associations between treatment delay and increased complication rates [18,19,20]. A meta-analysis performed by Foote et al. (2021) on 84 studies (18,239 patients) found an association between timing of debridement and surgical site infection (odds ratio [OR] = 1.29) [20]. On further analysis of Gustilo type-III injuries, Foote et al. found a progressive increase in risk of infection with critical time thresholds of 12 h (OR = 1.51) and 24 h (OR = 2.17). Importantly, all patients included in this audit required flap coverage, representing a particularly high-risk subgroup, and complication rates should be interpreted within this context.

Comparisons with published UK audits further emphasise the impact of geography and service structure on open fracture care. Lacey et al. audited BOAST4 compliance for open lower-limb fractures in a major UK trauma centre following the introduction of on-site plastic surgery [21]. Of 113 patients, 25% of high-energy injuries underwent initial debridement within 12 h, while 85% were debrided within 24 h. In comparison, only 6.67% of patients in our cohort were debrided within 12 h, and 46.7% within 24 h. Lacey et al. reported a mean time to definitive soft tissue closure of 77.4 h; however, only 38.9% of included patients required free flap coverage. By contrast, the mean time in our patient cohort was 194.5 h. Interpretation of these differences between the findings by Lacey et al. and our patients is limited by substantial differences in the geographical size of the serviced area, inclusion of upper limb injuries in our patient cohort, and lack of subclassification of time to free flap soft tissue coverage in the Lacey cohort. However, the comparison demonstrates a clear difference in compliance rates and length of time to surgical intervention, of which the aforementioned delays experienced by rural WA patients are partly responsible.

Aside from the BOAST4 guidelines, several international guidelines address the timing of surgical management of open fractures, including the NICE NG37 (UK) [22], AAOS (USA) [23], and EAST (USA) [24]. Both BOAST4 and NICE NG37 guidelines recommend initial surgical debridement immediately, within 12 h, or within 24 h, depending on injury classification, and definitive coverage within 72 h of injury. In contrast, AAOS and EAST do not specify an optimal timeframe for surgical intervention, instead recommending timely debridement within 24 h based on a case-by-case decision, and challenge the historical six-hour rule. Although multiple guidelines exist, their applicability to rural and remote patients in WA is limited by the unique challenges and extended transfer times inherent to this population. This gap could potentially be filled by either future Australia-based guidelines or adaptations to already existing guidelines to be context-specific for rural patients. Within which it would be prudent to highlight the importance of strengthening the relationships between rural/remote healthcare sites, transfer services, and the tertiary trauma centre, to continue to improve the quality of pre-transfer patient care. It is important to note that the purpose of context-specific guidelines is not to improve reported guideline adherence but to set achievable goals and to provide applicable recommendations that improve patient outcomes.

Importantly, the BOAst guidelines were developed within the United Kingdom healthcare context, which encompasses a substantially smaller geographical area than WA [25,26], and is serviced by a greater number of major trauma centres. The United Kingdom is serviced by 27 major trauma centres, compared with a single centre in WA (Perth only) [27]. These differences raise important concerns regarding the feasibility and appropriateness of applying BOAst timelines to rural WA. While the guidelines remain appropriate for metropolitan patients, a more pragmatic and context-specific approach is required for rural patients requiring long-distance transfer [28].

This study has limitations, including its retrospective design and small sample size, which limit statistical inference and preclude robust assessment of predictors of delay or complications. Unfortunately, several patients were lost to follow-up following the six-month period due to non-attendance, moving to private clinics, or being managed by community general practitioners. This study is also limited by incomplete data on known risk factors such as smoking, BMI, and comorbidities, which may confound the interpretation of complication rates [29,30]. Despite these limitations, this audit provides valuable insight into the practicality of, and real-world barriers to, timely open fracture care in rural WA.

## 5. Conclusions

The unique challenges experienced by rural and remote trauma patients in WA are representative of a more widespread issue faced by rural patients generally, whether in Australia or internationally. Geographic barriers have demonstrated significant delays, making it difficult to comply with established guidelines. This study highlights the need for guidelines that acknowledge the practical challenges of delivering timely care to rural and remote patients, alongside strategies aimed at minimising avoidable delays and optimising patient outcomes.

## Figures and Tables

**Table 1:** Demographics.

N = 15	Patients n (%)
Demographics	
Male	11 (73.3)
Age (Years: Median (IQR))	31 (23.15–41.5)
Indigenous	7 (46.6)
ISS (median (IQR))	13 (10–22)
Injury	
A	4 (26.7)
B	11 (73.3)
C	0
Injury location	
Hand	1 (6.67)
Arm/Forearm	1 (6.67)
Tibial plateau	1 (6.67)
Tibial shaft	7 (46.7)
Ankle	5 (33.3)

ISS: injury severity score. IQR: interquartile range.

**Table 2:** Acute and delayed complications occurring in the patient cohort.

N = 15	Patients n (%)	Injury Classification (n)
Acute complications	4 (26.7)	
Deep infection	3 (20)	A (2), B (1)
Fixation failure	0	
Flap failure	1 (6.7)	A (1)
Delayed complications	1 (6.7)	
Deep infection	1 (6.7)	B (1)
Fixation failure	0	
Flap failure	0	

## Data Availability

The original contributions presented in this study are included in the article. Further inquiries can be directed to the corresponding author.
